# A Rare and Interesting Case of a Massive Secondary Spinal Chondrosarcoma and Review of the Literature

**DOI:** 10.1155/2015/386482

**Published:** 2015-05-03

**Authors:** Anthony Adimonye

**Affiliations:** Sarcoma Unit, Royal National Orthopaedic Hospital NHS Trust, London HA7 4LP, UK

## Abstract

Chondrosarcoma, the second most common primary malignancy of the bone, is malignant cartilage forming tumour that very rarely involves the axial skeleton. It may arise as a primary bone tumour or as a secondary lesion from a preexisting benign cartilaginous neoplasm such as osteochondroma or enchondroma. A rare case of a massive secondary lumbar spine chondrosarcoma is presented. Management consisted of an initial posterior spinal stabilization and fusion and then a curative radical *en bloc* tumour resection. A review of the literature is also presented.

## 1. Introduction

Chondrosarcoma is the second most common primary malignancy of the bone, of which men are more often affected than women [1–5]. Chondrosarcomas may arise as a primary bone tumour or as a secondary lesion from a preexisting benign cartilaginous neoplasm such as an osteochondroma or enchondroma. They characteristically involve the bones of the pelvic girdle, shoulder, and the proximal ends of the femur and humerus [6–8]. Spinal chondrosarcoma is a rare occurrence [9–12].

A case of a male patient with a large secondary spinal chondrosarcoma arising from the malignant degeneration of a lumbar vertebral osteochondroma is presented and the inherent complex surgical management is discussed, in light of the literature.

## 2. Case Report

A 29-year-old male, warehouse worker, presented with a noticeable mass in the left side of his abdomen and weight loss of three and half stones over a four-month period. His past medical history and family history were unremarkable. Physical examination revealed a large, nontender, and fixed left-sided abdominal mass and a normal neurovascular examination except an isolated reduction in light touch sensation in the L2-L3 dermatomes of his left leg only.

**Figure 1:**
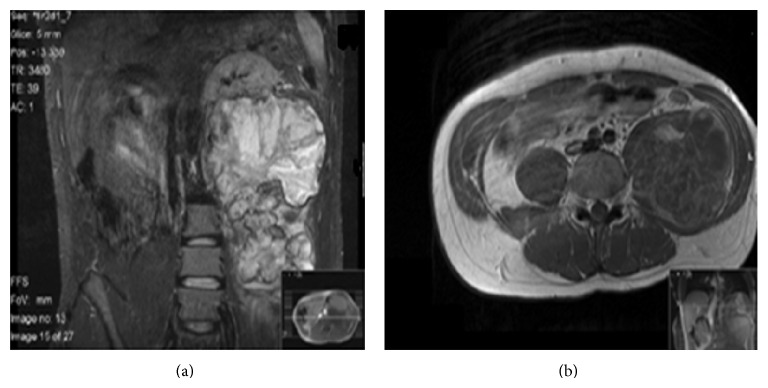
MRI scan (a) coronal view showing a large left lesion arising from the L3 vertebrae displacing the left kidney superiorly. (b) Axial view at the level of L3 depicting the lesion and its complete obliteration of the left psoas muscle.

**Figure 2:**
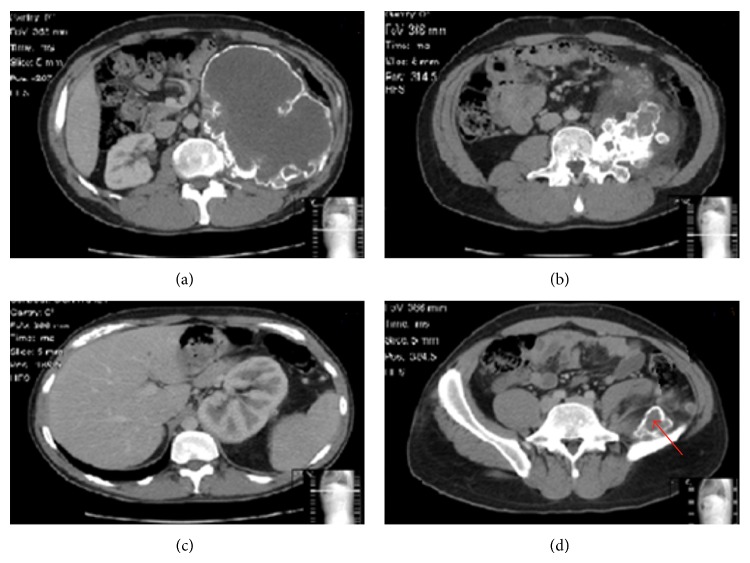
CT scan (a) axial view at L3 showing the distinct peripheral calcification of this large lesion and the displacement of intra-abdominal organs and blood vessels. (b) Axial cut through the middle third of the L3 vertebra depicting the L3 vertebral pedicle origin of the lesion with marked intralesional calcification. (c) Axial view at the level of T12 showing the superior displacement of the left kidney. (d) Axial view at the level of S1 showing the lesions inferior extension into the pelvis (red arrow).

An abdominal and pelvic magnetic resonance imaging (MRI) and computed tomography (CT) demonstrated a large, 19 × 16 × 11 cm mass arising from the anterolateral aspect of the L3 pedicle with peripheral calcification, completely obliterating the distal psoas muscle and causing a considerable mass effect to surrounding structures, with displacement of the left kidney. Distally, the tumour extended to the level of the pelvic inlet but not into the spinal canal (Figures 1 and 2).

CT guided biopsy of the left abdominal mass confirmed the diagnosis of a low-grade chondrosarcoma arising from an osteochondroma from the lateral body of the L3 vertebrae. The difficult anatomical location and the large size of the lesion greatly increased the complexity and risks of any surgical approach. In order to improve the chances of surgical success and reduce and prepare for possible risks and complication such as catastrophic haemorrhage, spinal instability, and paralysis/neurological deficit, a two-stage surgical procedure and a multidisciplinary team approach to a curative* en bloc* tumour resection were utilized.

In the first stage, a posterior midline incision to expose the lumbar spine was performed. Bilateral pedicle screws were placed at the levels of L2 and L4, with rods fixated at the end of the procedure for spinal stabilisation. Bilateral laminectomies and facetectomies were performed leading to exposure of the nerve roots, of which the left L3 nerve root was identified and ligated. A partial osteotomy was carried out at the level of L3 within the vertebral body to allow ease of hemivertebral body resection with the forthcoming* en bloc* tumour resection.

**Figure 3:**
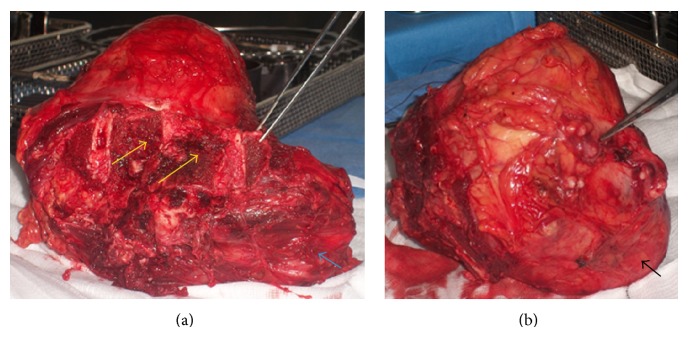
Gross specimen of the mass along with the partially excised vertebral bodies of L1 to L4 (yellow arrows: excised vertebral bodies, blue arrow: excised psoas major muscle, and black arrow: intact tumour envelope).

**Figure 4:**
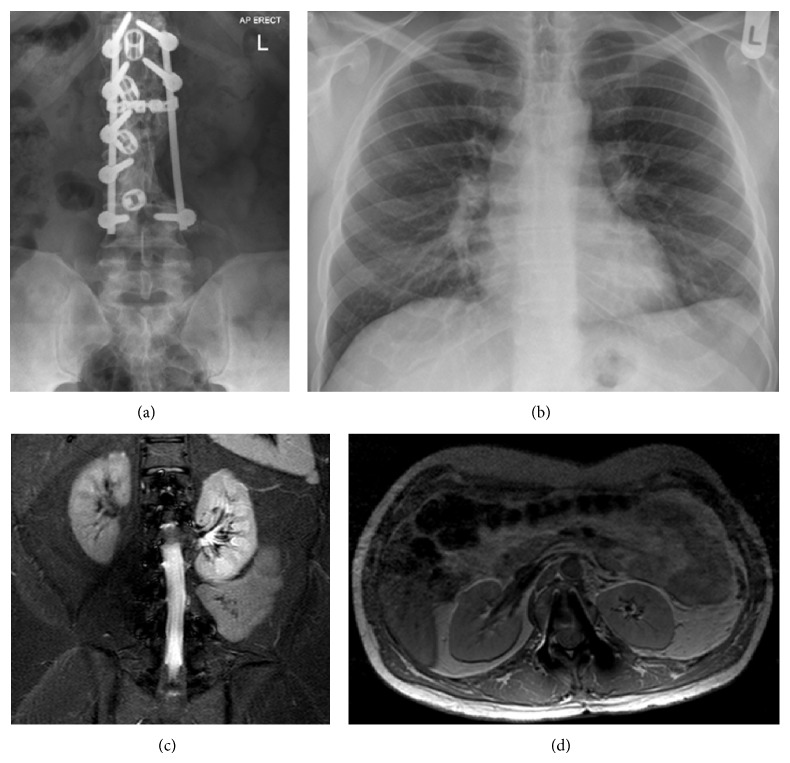
Five-year follow-up imaging (a) anteroposterior lumbar radiograph demonstrating stability of instrumentation. (b) Posteroanterior chest radiograph with clear lung fields. (c and d) Axial MRI at the level of L1 and coronal MRI with no evidence of macroscopic local tumour recurrence.

The second stage of the procedure involved an anterior thoracolumbar approach with the patient in the supine position. The intra-abdominal contents including the major vessels were identified and appropriately mobilised, allowing clear exposure and access to the left-sided tumour and the vertebral bodies of L1 to L5. The anterolateral aspects of the left ribs from 10 to 12 were resected to allow access to the superior part of the lesion. The tumour was resected* en bloc* with an intact envelope, surrounding soft tissue and completion of the L3 hemivertebral column resection osteotomy with partial resection of the vertebral bodies of L2 and to a lesser extent L1 and L4. After closure of the anterior wound, the patient was placed prone. The previous posterior midline wound was reopened and extended to expose the thoracolumbar spine from T12 to L4. The original posterior instrumentation was extended to include T12 and L1, with only right unilateral pedicle screws to L2 and L3 and bilateral pedicle screws to T12, L1, and L4. Spinal fusion from T12 to L4 using intervertebral titanium cages filled with allograft and demineralized bone matrix for additional stability was performed. The posterior needle biopsy tract was resected and the large excised tumour was sent for histopathological evaluation, which affirmed the previous biopsy results of a low-grade classical secondary chondrosarcoma with all margins free of tumour (Figures 3 and 4(a)).

Postoperatively, he had significantly reduced sensation in his L2-L3 dermatomes, due to the intraoperative sacrifice of his left-sided L3 nerve root, which was expected; otherwise he was well with no other symptoms. He was discharged after 3 weeks of hospital admission with a hard back and front brace for 6–8 weeks. At five-year follow-up, the patient had no new symptoms, local recurrence, or metastatic disease (Figure 4).

## 3. Discussion

Chondrosarcomas are a heterogeneous group of tumours, composed entirely of hyaline cartilage matrix and chondrocytes and range from slow-growing low-grade tumours to aggressive high grade forms [8, 13]. It is most commonly found in the long bones and pelvic girdle and less than 12% of all chondrosarcomas occur in the spine [14, 15]. They can occur at all spinal levels; however, most of them are commonly found at the thoracic level with lumbar spine involvement being relatively rare [9, 10, 15].

Primary chondrosarcomas, which account for two-thirds of all chondrosarcomas, arise* de novo* and are a tumour of adulthood and old age, with a peak incidence in the fifth and seventh decades of life. Secondary chondrosarcomas, which constitute the remaining one-third of all chondrosarcomas, are those that are superimposed on preexisting osteochondromas or enchondromas [16, 17]. This group of tumours occurs in a relatively younger group of patients, with the average age of diagnosis reported to be around 35 years [17–19].

Osteochondromas represent 30–40% of benign bone tumours and are the most common precursor lesions for chondrosarcoma. They can occur as solitary lesions or as hereditary multiple exostoses (HME), which is an autosomal dominant condition [7, 18]. Spinal osteochondromas are extremely uncommon, representing between 1 and 4% of all osteochondromas with cervical lesions being the most common, accounting for 56% to 80% of those occurring in the spine. Rarely do they arise from the lumbar spine [7, 8, 19].

The malignant transformation of an osteochondroma to chondrosarcoma is well described, albeit a rare occurrence. The conversion typically results in a low-grade chondrosarcoma, but higher grades are possible. This malignant conversion is normally associated with a sudden increase in the size of the lesion, increased or new-onset pain, an increase in the cartilaginous cap thickness, and recurrence of the lesion after total resection [7, 17–20]. The true incidence of malignant transformation remains unclear, as many osteochondromas and enchondromas are asymptomatic and are rarely identified [11, 17]. However, the risk of developing chondrosarcoma in a solitary osteochondroma is reported to be 1% and 10% for those with HME [16, 18]. To the best of our knowledge, only 3 other cases of a secondary lumbar spine chondrosarcoma arising from a solitary osteochondroma have been reported in the English literature [7, 21, 22].

Patients with spinal chondrosarcoma usually present with visible and/or palpable mass, pain, and variable neurological symptoms [2, 8, 10]. Our patient presented with no neurological symptoms and we found only a reduction in light touch sensation in his L2-L3 dermatomes. This was likely due to compression of the lumbar plexus particularly the lateral cutaneous nerve by the chondrosarcoma mass.

Typically, spinal chondrosarcoma has a malignant appearance radiographically. Plain radiographs usually demonstrate a radiolucent lesion with varying degrees of a cartilage type matrix calcification, usually a “*ring and arc*” calcification pattern [1, 14]. CT scans classically show osteolytic lesions with speckled calcification, aggressive growth from scalloping, and destruction to extension into soft tissue, all of which were evident in our patient's radiological imaging [14, 16]. Both MRI and CT scans are key components in preoperative planning stage for the surgeon, giving useful information about the tumour and its relationship with vital structures and the adjacent neurovasculature [4, 22]. In addition, MRI imaging is also the best modality for differentiating between chondrosarcoma and osteochondromas and also for evaluating the cartilaginous cap, which is an important criterion for the malignant transformation of osteochondromas [16, 20].

Secondary chondrosarcomas are diagnosed histologically but radiological and gross features of the tumour need to be taken into account and correlated. Spinal chondrosarcoma usually belongs to a mesenchymal or clear cell histological subtype. However, our patient displayed a low-grade conventional (classical) histological subtype. For secondary spinal chondrosarcomas there are two surgical options, the first of which involves complete* en bloc* excision with wide disease free margins. This is usually the treatment of choice, as this provides the potential for cure and a significant reduction in recurrence rates, which is said to be as low as 3 to 8% [16, 20, 22, 23]. The second is extensive intralesional curettage followed by local adjuvant chemical or thermal ablation and bone graft for select low-grade tumours confined to bone. And acceptable local recurrence rates and good functional outcomes have been observed with this technique [14, 20]. Chemotherapy and radiotherapy have proved largely ineffective in the treatment of spinal chondrosarcoma [4, 12, 22–24].

Complete* en bloc* excision for spinal chondrosarcomas is fraught with multiple technical difficulties such as the size of the tumour (which is normally larger in axial locations), anatomical constraints (e.g., major blood vessels and the spinal cord), risks of spinal instability, and inflicting new neurological deficits [1, 3, 12, 22]. All these factors result in the eventuality that complete surgical resection in the spine is not always possible; thus, surgical cure in this region is uncertain and local recurrences have been well documented and the risk of distant metastases increased [4, 20]. Due to these operative difficulties, chondrosarcomas of the vertebral column have a poor prognosis independent of histological grading or subtype, with 5-year survival rates between 25 and 54% compared to 87% for chondrosarcoma generally [14, 23, 25]. However, in the likelihood of incomplete surgical excision with insufficient margins or local recurrence, the use of adjuvant radiotherapy and particle beam therapy has been implemented with some degree of success with 10-year survival rates of up to 86% reported [12, 23, 26].

In our case, we were able to achieve a complete* en bloc* resection with adequate tumour-free margins and at one-year follow-up no sign of recurrence was noted. However, patients with chondrosarcoma may have a delayed course of local recurrence or metastatic disease and long-term follow-up is advised [1, 14].

## 4. Conclusion

Spinal chondrosarcoma is a rare occurrence and this report depicts a rare and interesting case of the surgical management of a grossly enlarged secondary spinal chondrosarcoma arising from the malignant transformation of a lumbar vertebral osteochondroma with a favourable outcome.
